# Capacity Building in Global Mental Health: Professional Training

**DOI:** 10.3109/10673229.2012.655211

**Published:** 2012-02-15

**Authors:** Gregory L Fricchione, Christina P C Borba, Atalay Alem, Teshome Shibre, Julia R Carney, David C Henderson

**Affiliations:** 1 Harvard Medical School and Pierce Global Division, Department of Psychiatry, Massachusetts General Hospital, Boston, MA; 2 Department of Psychiatry, Addis Ababa University

**Keywords:** capacity building, global mental health, psychiatry education

## Abstract

We suggest that the optimal approach to building capacity in global mental health care will require partnerships between professional resources in high-income countries and promising health-related institutions in low- and middle-income countries. The result of these partnerships will be sustainable academic relationships that can educate a new generation of in-country primary care physicians and, eventually, specialized health professionals. Research capabilities will be an essential educational component to inform policy and practice, and to ensure careful outcome measurements of training and of intervention, prevention, and promotion strategies. The goal of these academic centers of excellence will be to develop quality, in-country clinical and research professionals, and to build a productive environment for these professionals to advance their careers locally. In sum, this article discusses human capacity building in global mental health, provides recommendations for training, and offers examples of recent initiatives. (Harv Rev Psychiatry 2012;20:47–57.)

A new movement has emerged to bring attention to the importance and feasibility of increasing mental health capacity in low- and middle-income countries (LAMICs). The case for psychiatric training and care as an integral part of the global health agenda is well articulated in the *Lancet* series on global mental health, published in 2007 and 2011; these two series of articles echoed the U.S. surgeon general's 1999 report on mental health, with its emphasis on mental health as an essential component of general health care,^1^,^2^ and catalyzed a Movement for Global Mental Health.^3^ These reports underscore that the mental disorders have a tremendous impact on mental wellness and that this impact, in turn, substantially affects other health indicators—evidenced by increased risks of cardiac disease, stroke, and diabetes.^4^–^8^ The publications also highlight that mental illnesses contribute to mortality through suicide and through worsening of medical diseases. With the emergence of successful studies of pharmacological and psychological treatments for mood disorders and schizophrenia in resource-constrained settings, the need to improve mental health care in all areas of the world becomes even more salient.^9^–^12^

## THE PROBLEM OF CAPACITY AND RESOURCES

Four hundred and fifty million people around the world suffer from mental disorders,^13^ and a significant part of the global burden of disease is attributed to the 25% lifetime prevalence of mental diseases.^14^ One reason for the new focus on global mental health is the development of an important research measure called *disability-adjusted life years* (DALYs).^15^ This measure refers to the “sum of years of potential life lost due to premature mortality and the years of productive life lost due to disability” (http://www.who.int/mental_health/management/depression/daly/en/).^15^–^17^ Mental illnesses, which are highly disabling, rise in importance when disability is taken into account. They constitute 14% of the overall burden of disease (as measured in DALYs) and 28% of the noncommunicable disease burden.^13^ They also account for a third of the years lived with disability by adults globally, and are five of the ten leading causes of disability.^14^

In 2001, the World Health Organization (WHO) issued a *World Health Report* focusing on mental health.^13^ It suggested the following solutions to the problems of global mental health:

- provide treatment in primary care
- make psychotropic medications available
- give care in the community
- educate the public
- involve communities, families, and consumers
- establish national policies and legislation
- develop human resources
- link with other sectors
- monitor community mental health
- support more research

The landmark *Lancet* global mental health series of 2007 and 2011, along with WHO's *Atlas: Mental Health Resources in the World*, highlighted the major shortages of psychiatrists, psychiatric nurses, psychologists, and social workers in LAMICs.^18^–^20^ These shortages reduce treatment and care opportunities.^18^ For example, the median rate of psychiatrists in low-income countries is .05 per 100,000 population, versus 8.59 psychiatrists per 100,000 population in high-income countries^19^ (HICs)—which means that the median number of psychiatrists in HICs is 172 times greater than in low-income countries.^20^ Consequently, nearly half the world's population resides in countries where one psychiatrist is available to serve 200,000 people or more.^19^ Similarly, while nurses constitute the largest workforce in the mental health system, the gaps in resources remain significant, with .42 psychiatric nurses per 100,000 population in low-income countries versus 29.15 nurses per 100,000 population in HICs. The median rate of nurses ranges from .61 per 100,000 in Africa to 21.93 (per 100,000) in Europe.^19^

Training is essential to develop a workforce to meet existing need, but psychiatric training programs remain inadequate for that purpose. In a study of primary care training in 37 LAMICs (based on data extracted from published country profiles), 61% of countries reported the integration of psychiatry and behavioral sciences teaching into their undergraduate curricula, 20% reported the integration of assessment and treatment protocols for mental disorders in primary care, 17% described some integration between primary care and mental health services, 27% reported mental health retraining for primary health care physicians, and 13% had refresher programs for primary health care nurses.^21^ It must be noted, however, that data about mental health refresher training of nonphysicians and about collaborations between physicians, nonphysicians, primary health care staff, and traditional healers were available in less than 10% of profiles studied.^21^

This critical caregiver shortage is especially apparent in sub-Saharan Africa, where the availability of mental health care is radically limited because of the inadequate numbers of mental health professionals.^18^ Liberia, which experienced almost two decades of violence, has one psychiatrist for a population of approximately 3.5 million people. Likewise, Chad and Eritrea, both affected by serious conflicts, have only one psychiatrist for their populations of 9 million and 4 million, respectively. Afghanistan (population of 25 million), Rwanda (8.5 million), and Togo (5 million) each has two psychiatrists.

Brain-drain issues contribute greatly to the mental health capacity problem in LAMICs. Like other health professionals in these countries, mental health providers often migrate to areas with higher incomes. The lack of education and training opportunities in mental health care also severely handicaps LAMICs, as they have no means to make up for the scarcity of mental health professionals from an internal source. Limited financial resources complicate matters. Close to a third of countries have no specified mental health budget, and of the 101 countries that have a mental health budget, most spend less than 1% of their total health budgets on mental health care.^18^ Despite the burden of disease being relatively high in low-income countries, they spend the least on mental health, when measured as a proportion of their overall health budgets. A third of low-income countries provide mental health care only through out-of-pocket payments.^18^ The result of this bleak assessment is that residents in countries with the highest need have the poorest access to care.

The scarcity of mental health resources and inequalities in access to them have significant consequences, the most important of which has been referred to as the *treatment gap*.^18^ This expression refers to the proportion of those who need but do not receive care: for example, as many as one out of three individuals (worldwide) with schizophrenia do not receive any treatment. The treatment gaps for depression and dysthymia, bipolar illness, and anxiety disorders are all greater than 50%.^22^ The challenge presented here is most pressing in poorer countries. WHO reports that the treatment gap for serious disorders is 76%–85% in LAMICs, whereas it is 35%–50% in developed countries.^23^ Even when some treatment is provided, it is often less than optimal and therefore ineffective. The result is an untold amount of human suffering, disability, and economic loss.^18^

The modest availability of psychiatric medications globally is reflected by the WHO List of Essential Medicines, which includes only three antipsychotics (chlorpromazine, fluphenazine, and haloperidol), two antidepressants (amitryptiline and fluoxetine), and one anxiolytic (diazepam).^24^ These medicines are often not available in LAMICs. Notably, the list is confined to first-generation antipsychotics and omits the newer generation of antipsychotics available in the developed world.^24^

Another problem concerns the low numbers and limited types of health workers trained and supervised to provide mental health care in LAMICs. There are many possible reasons for this situation.^25^ The working conditions in public mental health services are poor, with a lack of incentives to work outside of cities. Professional biases operate against expanding the roles for nonspecialists within the mental health workforce. Medical students and psychiatric residents are often trained in psychiatry only in stand-alone mental hospitals, making it difficult to translate their education into primary care settings. Mental health specialists are forced to spend time on the front lines providing care rather than becoming teachers and supervisors. The infrastructure for building community-based supervision in mental health care is lacking. Mental health leaders often do not possess the skills and experience necessary to move public health opinion; they are often clinicians without public health training and are overburdened by clinical and management responsibilities, as well as by private practices they have established to make ends meet. There have been calls to upgrade the number and quality of trained mental health workers, to train and oversee primary care staff to provide referral capacity, and to provide ongoing supervision and support for primary care systems in mental health.

It is clear that the world has a mental health problem. The responsibility for mental health care transcends national borders, race, ethnicity, culture, class, gender, and the distinction between high- and low-income nations. The development of mental health capacity, particularly in LAMICs, requires collective action based on local and global partnerships. A major limitation in implementing a solution to mental health problems is the lack of trained and skilled professional mental health caregivers. There is a critical need for HIC mental health professionals to collaborate and serve in many LAMICs, given the lack of human capital in these countries. While we recognize that training community workers or health extenders may be an important step in building capacity, we also suggest that there is a need to develop mental health leadership and to train high-level health professionals. In this context, HICs can help address global mental health problems by developing caregivers, teachers, and researchers falling into the following categories:

- *Global mental health psychiatrists*. These psychiatrists would complete a psychiatry residency program, followed by a one- to two-year fellowship in global psychiatry or global psychiatry research. They might also obtain a master of public health degree (MPH) to help with their public health and policy missions. The contribution of the HIC academic medical centers would be to institute training programs for these professionals, who could then serve as the crucial professional personnel, as both faculty and colleagues, needed for LAMICs' efforts to build capacity and to sustain evidence-based mental health services. Fellows would be trained to conceptualize mental health policies and plans; develop and evaluate mental health systems; develop and manage international research teams; build productive international collaborations; work with government, nongovernmental organizations, and academics abroad; and develop research questions that are clinically and culturally relevant to identified community and public health needs.
- *Global mental health psychologists and social workers*. Psychologists would complete their doctoral-level degrees and internships, and then pursue postdoctoral education in global mental health or global mental health research. For other students and trainees, global mental health tracks could be established within master of social work degree (MSW) programs, and other options for postgraduate training are also possible; a good example would be the Global Mental Health Trauma and Recovery Certificate Training Program offered by the Harvard Program in Refugee Trauma.^26^ Here, again, the contribution of HIC academic medical centers would be to institute training programs for these professionals, who could provide the crucial psychosocial services, and serve as the faculty and colleagues, needed for LAMICs' efforts to build capacity and to sustain evidence-based mental health services.
- *Global mental health psychiatric clinical nurse specialists*. These specialists could have career opportunities built into their training that would equip them to work more effectively in LAMICS. Their skills would be of great assistance since much of the primary care in rural settings is the responsibility of nurses and similar health workers. Academic nursing programs in HIC academic medical centers may make an important contribution by creating and maintaining such educational opportunities.
- *Global mental health training for emergency and primary caregivers*. A major role of the specialists described above—after they have graduated and had overseas experiences—would be to educate their HIC academic medical center colleagues in emergency medicine, nursing, and primary care medicine in their preparation for overseas work. In addition, when these specialists trained in mental health are themselves deployed to LAMICs, they would have the skill sets to provide mental health training and supervision to LAMIC colleagues in medicine, nursing, and other health fields, as well as mental health training and supervision for lay caregivers, including traditional healers.

The approach to building a mental health infrastructure for LAMICs must be bidirectional. In addition to nurturing career trajectories in academic medical centers residing in HICs, efforts need to be made to develop a cadre of home-grown, in-country professionals and basic mental health workers. Linking these efforts is critical. Against this background we will briefly review a strong effort currently taking place in sub-Saharan Africa. We should also mention that one significant benefit of training HIC mental health professionals with global experience is that their skills and experiences from abroad will likely improve the care of their patients at home, who are becoming more culturally, racially, and ethnically diverse. Likewise, the experience abroad in system evaluation, development, and innovation will also inform professional work and programs in the HICs themselves.

## THE ETHIOPIAN EXPERIENCE

Ethiopia, located on the horn of Africa, has a population of 88 million in an area the size of Texas. The country continues to experience many disasters, including drought and famine, high rates of human immunodeficiency virus infection, tuberculosis, and malaria, internal displacement due to civil and border wars, grinding poverty, and other stressors and traumas. It is one of the world's poorest countries: the gross national income per capita is just U.S.$1190, and 23% of the population earn less than U.S.$1 per day.^27^ Its challenges in the area of mental health are emblematic of what many low-income countries face. It is therefore helpful to examine the recent attempts to address these issues—including the important strategies of *twinning* and *task shifting*, which are both described below.

### Psychiatry and Mental Health Resources

The WHO's *Atlas of Mental Health Resources*, published in 2005, indicates that Ethiopia had no mental health policy, national mental health program, community care in mental health, substance abuse policy, or applicable mental health law.^28^ It is promising that a mental health policy for Ethiopia has now been written and accepted. An obvious barrier to Ethiopian mental health care is the low number of psychiatrists and psychiatric nurses, along with the complete absence of psychologists or social workers. Many other obstacles affect access to psychiatric care. Since the majority of Ethiopians believe that the manifestations of psychiatric disorders are due to spiritual causes, they first seek out traditional healers for emotional and behavioral problems.^29^,^30^ These practitioners' methods include herbal remedies, holy water, “fumigation” with incense, exorcisms, and other rituals. Only when such methods fail might families seek modern psychiatric treatment for their family members, often months to years after the onset of difficulties.

Even for those who seek out modern psychiatric treatment, resources are limited. Psychiatry was introduced into the medical school curriculum in Ethiopia in the late 1960s.^31^ In 2000, the country's nine psychiatrists had all been trained in Europe or North America. Over the years, three of these nine psychiatrists were hurt and almost lost in travel accidents as they moved from one part of the country to another in an effort to supervise other caregivers. The first psychiatry residency program (described below) was established in 2003 in Addis Ababa, the capital. Seven residents were accepted in the first residency class. Three faculty members taught these young psychiatry residents in a three-year psychiatry program.

As of 2007, there were 33 psychiatrists in the country, the majority of whom were practicing in Addis Ababa.^32^ In addition, psychiatrists now man departments in five medical schools—at Addis Ababa, Haromaya, Hawassa, Jimma, and Mekele universities—with more departments being planned. A central role for these psychiatrists is to help educate primary care physicians who will provide mental health care in their practices. At present, psychiatric nurses stationed at regional and district hospitals provide the majority of the mental health care outside of the capital region.^30^ A children's hospital is soon due to open in Addis Ababa, which will include a 20-bed inpatient unit and an outpatient service. At present, however, only three psychiatrists do child psychiatry in Ethiopia; many more are needed.^33^

To help provide a significant portion of the formal and clinical teaching for the psychiatry residency program at Addis Ababa University, the Departments of Psychiatry at that university and the University of Toronto initiated the collaborative Toronto Addis Ababa Psychiatry Project (TAAPP; http://www.utoronto.ca/ethiopia/) in 2003.^34^ The participation of the University of Toronto's Department of Psychiatry is an outstanding example of the *twinning process*—a long-term, committed partnership of institutions and communities that provides peer-based technical assistance to achieve common goals through the sharing of ideas, labor, and risks. Underlying the concept of twinning is the notion that in facing common problems together and by joining forces, we can accomplish more than we ever could alone. TAAPP assembles teams of two staff psychiatrists and one psychiatry resident, who travel to Addis Ababa to provide one month of teaching and clinical supervision for the Ethiopian psychiatry residents. During the first three years of the program, three trips were made each year, and each trip focused on teaching specific topics and areas as determined by the Ethiopian curriculum—for example, child psychiatry, mood disorders, psychotic disorders, and psychotherapy. In 2006, the first seven Ethiopian residents graduated, with several joining the faculty of the Department of Psychiatry at Addis Ababa University. Subsequently, the frequency of TAAPP trips was reduced to twice per year, with the objective of enabling the new graduates to take on more of the teaching and educational leadership roles in the residency program.^34^ A one-year fellowship program was also started in Toronto for Ethiopian junior faculty.

**Figure 1:**
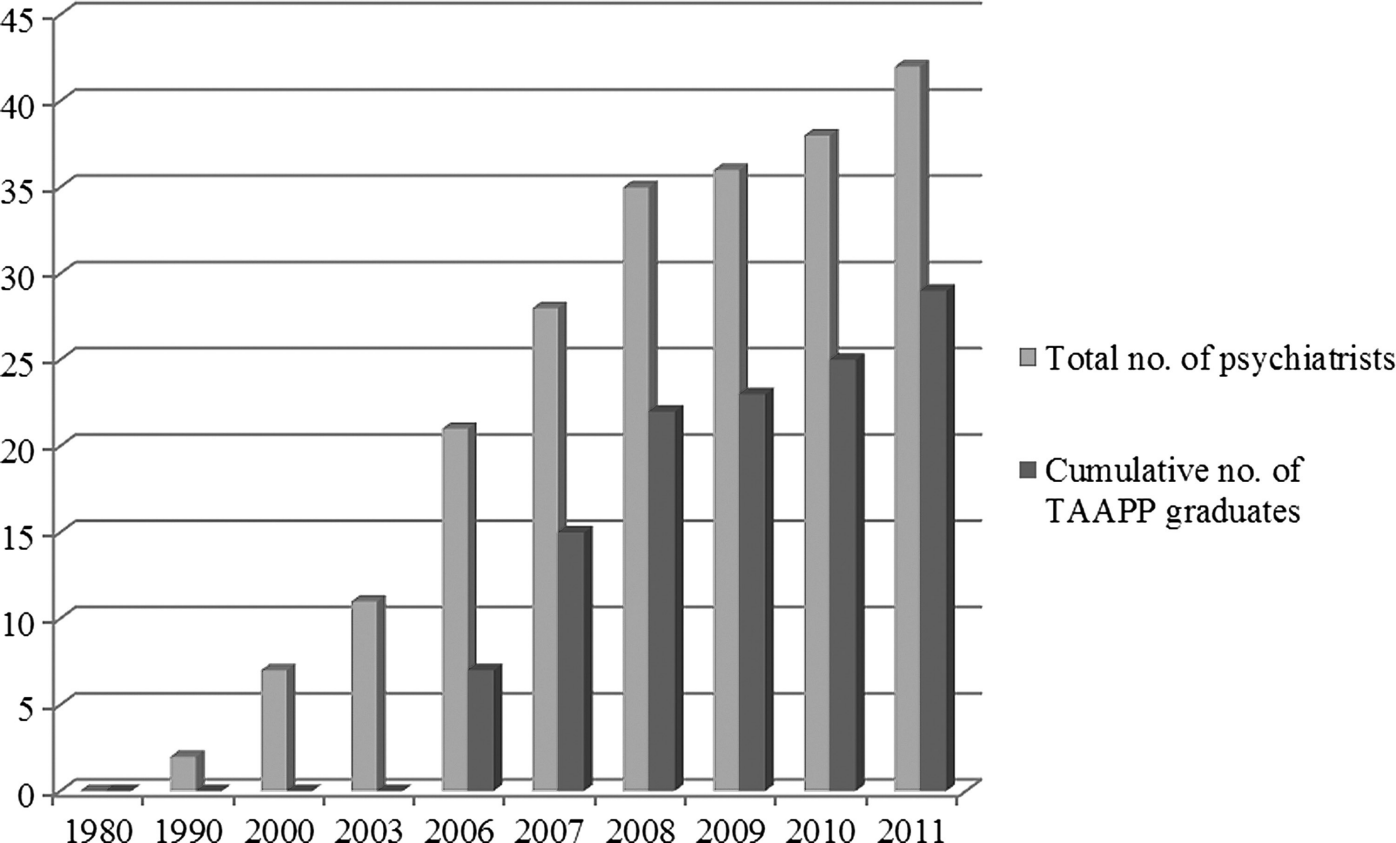
Ethiopian psychiatrists. Ethiopian population ∼ 88,000,000. Psychiatrist/population ratio in 2001 ∼ 1:2,000,000. TAAPP, Toronto Addis Ababa Psychiatry Project. Source: Department of Psychiatry, Addis Ababa University.

Between November 2003 and March 2010, TAAPP sent 17 teaching teams to Ethiopia. Through this exchange, a total of 28 Ethiopian psychiatrists has been trained, and the University of Addis Ababa psychiatry faculty has increased in size from 3 to 11. Four of Ethiopia's newly graduated psychiatrists have opened the country's first psychiatry departments in university hospitals outside the capital city of Addis Ababa. Perhaps most importantly—thanks in large part to the lobbying activity of Ethiopia's developing psychiatric community—mental health is now being integrated into the Ethiopian government's primary health care strategy. See Figure 1.

Now in its eighth year, TAAPP has expanded its activities to include training in Canada for new Ethiopian faculty members, support for new departments of psychiatry outside Addis Ababa, and the development of annual continuing education workshops for Ethiopia's expanding community of qualified psychiatrists. TAAPP also offers a model for expanding postgraduate training for other Ethiopian medical residency and PhD programs. It was the first of the twinning projects organized under the Toronto Addis Ababa Academic Collaboration (TAAAC; http://www.taaac.com), an umbrella organization that now includes six current educational partnerships between departments and divisions in six Faculties at the University of Toronto and their counterparts in Ethiopia. The purpose of TAAAC is to help Addis Ababa University build and strengthen capacity and sustainability in medical specialties and other health and nonhealth professional fields. It is an outstanding example of what a commitment from an academic medical center global psychiatry unit—funded on a shoestring—can accomplish when partnered with a university or medical center in a LAMIC. In 2009, in an initiative inspired by TAAPP, neurologists at the Mayo Clinic and Addis Ababa University collaborated to establish a neurology residency training program.

### The Ethiopian Public Health Training Initiative

In 1997, the Carter Center (http://www.cartercenter.org) and the Ethiopian government established the Ethiopia Public Health Training Initiative (EPHTI), which emerged from discussions between former U.S. president Jimmy Carter and Ethiopian prime minister Meles Zenawi. EPHTI had two major objectives: (1) to strengthen the teaching capacities of the public health colleges in Ethiopia through education of their teaching staffs, and (2) to collaborate with Ethiopians in developing curricular materials specifically created to meet the learning needs of the health center team personnel. Modules to educate public health workers have been produced on diverse topics in communicable and non-communicable diseases, preventive medicine, nutrition, maternal and child health, and family planning.

In May 2002, the EPHTI Council of Ethiopia approved a program to establish a mental health module for training health workers in Ethiopia. This module, produced by a technical committee of Ethiopians, focuses on common mental illnesses and on the severe illnesses and conditions identified by the WHO Mental Health Global Action Programme. This mental health training module is now used in Ethiopia's public health colleges to educate nurses and other health workers in mental health management—an example of the principle *of task shifting*, the process by which tasks are delegated to less specialized health workers to improve care coverage using existing human resources.

EPHTI nurses and health workers are trained to work in the 35 primary health care units (PHCUs), each responsible for a population of 100,000 people, and each staffed by two psychiatric nurses. A primary hospital, health center, and health posts form a PHCU, with each health center having five satellite health posts. These PHCUs are visited on a routine basis by the country's psychiatrists, who consult with the psychiatric nurses and related health workers on difficult cases. The psychiatric nurses and health workers pass that information to the appropriate clinicians and are also charged, more generally, with educating primary care physicians, nurses, and other health workers about mental health.

The Ethiopian story is an important one for global psychiatry. Providing good mental health services for a poor population in a developing country like Ethiopia requires a bi-level approach in which both psychiatrists and psychiatric nurses are trained, and also more bachelor's-level nurses and health officers. Building psychiatry resources by establishing an in-country psychiatry residency is an important step. It offers a modicum of protection against the brain drain that often occurs when doctors train overseas, and it provides much-needed manpower with professional expertise. To its credit, the University of Toronto has built a twinning relationship with Addis Ababa University; in so doing, the two collaborating psychiatry departments have made the Ethiopian residency a reality. One hopes that this model could be replicated, thereby connecting other university departments in the developed and developing world.

At the same time, it is important to remember that mental health professionals need to be educated at all levels, and on an ongoing basis, to maximize treatment access and to create a well-balanced, effective system of care. In Ethiopia, the development of a cadre of caregivers at the bachelor's level was made possible through EPHTI, the joint program of the Ethiopian government and the Carter Center. In addition, a new master's-level training program in integrated clinical and community psychiatry for midlevel health workers has been developed at Jimma University in its Department of Psychiatry and at Gondar University in collaboration with Amanuel Psychiatric National Referral Hospital. The program builds on the legacy of TAAPP and EPHTI. Likewise, building on its EPHTI experience, the Carter Center has begun a replication program in Liberia to develop a program to educate nurses and health workers in mental health care.

## BUILDING MENTAL HEALTH CAPACITY IN THE TWENTY-FIRST CENTURY

The Ethiopian experience suggests numerous strategic opportunities for international mental health partnerships, all of which will progressively become realities as the field of global psychiatry matures. In this regard, the enormous challenge of building resources and capacity for providing basic mental health services to LAMICs is going to require a revolution in medical education, both here in the United States and in other HICs, and in those very same LAMICs. This challenge opens up a much larger topic: how shall we educate clinicians in the twenty-first century?

Recently, a blue ribbon panel met to discuss new paradigms for educating health professionals for the twenty-first century. This effort was called *Education of Health Professionals for the 21st Century: A Global Independent Commission*^35^ (Commission). The Commission, comprising 20 professional and academic leaders from diverse countries, developed a shared vision and a common strategy for education in medicine, nursing, and public health. In its effort to analyze the existing barriers to global health care, the Commission took a global perspective, complemented by multiprofessional and systems approaches.

The Commission found that there are 2,420 medical schools, 467 schools with departments of public health, and many post-secondary nursing schools around the world.^35^ Four countries—Brazil, China, India, and the United States—each have over 150 medical schools; 36 countries have no medical schools; and 26 of the countries in sub-Saharan African each have 1 or no medical school.^35^ The resulting disparities in health care provision are profound, and the medical school distribution is obviously not aligned well with either populations or the national burdens of disease.

The Commission proposes several reforms.^35^ They recommend adoption of competency-based curricula, the promotion of inter-professional and trans-professional education that breaks down professional silos and enhances collaboration, the exploitation of information technology to promote learning, and adapting locally but harnessing resources globally. An overarching goal is to develop the capacity to be flexible in addressing local challenges while using global knowledge and experience. Opportunities for distance learning and the development of international exchange programs would significantly enhance the impact of the proposed reforms. More broadly, moving past the standard concept of academic *centers* to that of academic *systems* is a crucial step in developing the types of programs and interventions envisioned by the Commission. The settings used in existing health care education programs will need to be expanded, and primary care settings, in particular, will need to be used more extensively as sites of learning. Alliances and collaborations between educational institutions, governments, and nongovernmental organizations (included here are twinning relationships between universities in HICs and in LAMICs) will be essential for maximizing effectiveness. The Commission also notes that the reforms will require that leadership be mobilized, that investments be made, that accreditation across countries be aligned, and that global learning strategies be strengthened.

### Professional Training

An abundance of evidence indicates that capacity building is a major requirement for making progress in global mental health care. To focus on the United States for the moment, it becomes clear that we need more U.S. psychiatrists, psychologists, social workers, and public health and health policy specialists at the international level who have specialized training and skills in knowledge transfer, capacity building, policy, clinical training, and research.

One hopeful sign in psychiatry, at least, is that graduating medical students applying for entry into psychiatry residencies are beginning to request overseas experience and a global focus within the residency curriculum. Here at the Massachusetts General Hospital (MGH), approximately 30% of applicants seek out information about our Division of Global Psychiatry and want to know how they will become more proficient in global mental health during their training. When we began the division in 2003, the Harvard Program in Refugee Trauma already had an established place in our department, and a meeting of psychiatrists of African descent from all over the world—the “African Diaspora” meeting of November 2002—had just taken place at the hospital. In 2005, the philosophy behind our decision to establish the division was set out in an article in *Academic Psychiatry*^36^ The division's work benefits from a rich environment that includes the resources of Harvard Medical School, the Department of Global Health and Social Medicine, and the more recent experience of the Harvard University Institute for Global Health and the Harvard Humanitarian Initiative. In addition, MGH has itself recently started a Center for Global Health.

In the spirit of its visionary namesake, the mission of the Chester M. Pierce, MD, Division of Global Psychiatry is to make clinical, educational, and research contributions to world mental health and to help reduce the global burden of disease by learning from our neighbors and by contributing what we know to relieve the suffering from mental illnesses around the world. Residents in the MGH psychiatry department are permitted to arrange a monthlong elective during their community rotation in postgraduate year (PGY) 3, subject to careful advance planning with the directors of the division and the residency program. In addition, if the resident develops a sound plan of scholarship, he or she can spend three months of elective during PGY-4 in an overseas location. During PGY-3 a resident may also apply to become the chief resident in global psychiatry during PGY-4. This position can, if desired, be combined with the equivalent position in community psychiatry. Our residency program's support for global psychiatry training has likely been a recruitment bonus for us, and it has also allowed us to develop an innovative curriculum with the collaboration of some international partners.

### A Global Mental Health Psychiatry Residency Curriculum

Early in the life of the MGH Division of Global Psychiatry, it took part in a 2004–08 project funded by the U.S. Department of Education: the goal of this IMPACT program was to develop an international public mental health leadership training curriculum for psychiatry residents. Our collaborators included the Dalhousie University Department of Psychiatry (Canada) and the National Institute of Psychiatry—Instituto Nacional de Psiquiatría Ramón de la Fuente (Mexico). McMaster University (Canada) and the National Institute of Neurology—Instituto Nacional de Neurología y Neurocirurgía (Mexico) also served as training sites for the project. This consortium of institutions enabled resident psychiatrists from the three countries to complete a program that increased their knowledge of North American public mental health systems and sought to prepare them for leadership roles in international psychiatry. The innovative, three-month curriculum—which focused on how to advance current public mental health care practices in Canada, Mexico, and the United States—was prepared by faculty with help from residents at the six institutions. Under the program, residents spent three consecutive months at IMPACT training sites in Canada and Mexico and at MGH.

The program's specific curricular goals were to teach residents aspects of international and public sector mental health care in the following domains:

- mental health policy, plans, and programs
- mental health legislation and human rights
- mental health services
- mental health promotion and illness prevention
- mental health advocacy
- mental health human resources and training
- global mental health research
- essential psychotropic medications and ethnopsy-chopharmacology
- special topics such as trauma, refugee, and disaster psychiatry

Additional goals included developing leadership skills in the field of global psychiatry and fostering strong ties for further collaboration in the field. The overarching curriculum involved all three countries (Canada, Mexico, and the United States), with specific components being assigned and taught at each site. At each site we also took advantage of the expertise that was available, and commissioned special lecture and discussion sessions with prominent psychiatrists. The grant, now completed, had a permanent impact on the MGH/McLean Adult Psychiatry Residency Program's global mental health curriculum, which we have also made available (either web based or on CD-ROM) to other institutions in all three countries.

A key theme that ran through the IMPACT project and that guides our Division of Global Psychiatry is *bidirectionality*. When we interact with colleagues, trainees, and students in other countries and settings around the world, we stand to gain as much as we impart to others—and sometimes even more. The successful education of a cadre of young U.S. professionals requires interactions with sustainable mental health programs in academic systems in LAMICs, which will require educational models adapted to those settings—and not just standard U.S. models of training.

## A STRATEGY FOR CAPACITY BUILDING: SUMMARY

The development of global health institutes and global mental health divisions within academic medical centers and medical schools provides a framework for capacity building. These institutes and divisions can begin to take responsibility for coordinating educational efforts across a matrix of disciplines and stages of training. Representatives of medicine, dentistry, nursing, psychology, social work, and public health interested in the global health agenda can, in concert, begin to elaborate competency-based curricula that deliver the knowledge base of global health and mental health at each stage of training and that promote interprofessional and trans-professional education that breaks down professional silos and enhances collaboration. These efforts can then take advantage of information technology to make scalable the training and education needed to create more effective health professionals in LAMICs. Adapting such efforts locally to the areas of care most needed, while harnessing global knowledge and experience, will be essential, as will communicating the need to be flexible in addressing local challenges, even while using those same global resources. The best way to accomplish these goals may be to form twinning relationships between HIC institutions and colleges, medical schools, and other institutions of higher learning in LAMICs—not only to exchange information but to train and certify professionals capable of delivering evidence-based care in LAMICs at every level of care and in every area of need:

*Step 1:* HIC medical school/academic medical center establishes a *global health institute* or *center*.
*Step 2: A global psychiatry and mental health division* is formed within the global health institute. Educational programming establishes multidisciplinary career trajectories in global mental health professions and builds a cadre of trained and experienced clinicians, educators, and researchers equipped to work in LAMICs. An example of a career trajectory that started with subspecialty fellowship training supported by the U.S. National Institute of Mental Health is psychosomatic medicine. It is now a boarded subspecialty in psychiatry. One might expect that if fellowships in global psychiatry find support, a similar process culminating in a formally recognized subspecialty would emerge. It would likely include elements of psychosomatic medicine, given the interactive effects of mental and physical illnesses around the world.^37^
*Step 3:* The global psychiatry and mental health division forms a *twinning relationship* with a university, medical school, or public health school in a LAMIC, with the aim of establishing a center of excellence that will include training in psychiatry/mental health for physicians (for psychiatry residents and as part of the training for primary care physicians), nurses (for psychiatric nurses and as part of the training in basic nursing), psychologists (at the bachelor's, master's, and doctoral levels), social workers (at the bachelor's and master's levels), and public health specialists (at the bachelor's and master's levels). The urgent problem of child mental health in LAMICs can also be addressed using this model. Indeed, given the enormous challenge to provide mental health care to children globally, Belfer^36^ recommends establishing regional centers of excellence. Components would include resource libraries, access to psychiatric consultants, support, training, and clinical diagnostic functions. The goal would be to produce a sufficient number of adequately trained and culturally knowledgeable child mental health professionals to treat children and adolescents within the region.
*Step 4:* In addition to clinical training and certification, *research center development* should be part of the center of excellence so that sustainable research training can be developed within the LAMIC. The presence of a research center and trained researchers will help develop the knowledge base required to determine what evidence-based care is needed in that particular LAMIC and will also provide the basis for monitoring the outcomes of the center of excellence itself. Kleinman and Han^38^ have written that research is essential to improve the health of populations, to address the global burden of disease, to organize and fund appropriate systems of mental health care, to improve outcomes, and to reduce disability.

Studies in these mental health research centers would include the perspectives of public health, anthropology, epidemiology, and psychology, as well as psychiatry. Additionally, research would include training in cross-cultural methodologies. Research on mental illnesses demonstrates the presence of cultural variations in how these disorders manifest themselves.^39^ Since most epidemiological surveys utilize Western assessment tools and focus on diagnostic categories, important information is lost about how illnesses might appear in different cultural settings.^40^ Researchers have noted the importance of the following key steps in cross-cultural epidemiological work: (1) to consider the relevant anthropological and ethnographic material when designing studies, (2) to develop glossaries and operational definitions for symptoms and diagnostic categories to be studied, (3) to derive symptom patterns using multivariate analytic techniques rather than relying exclusively on a priori diagnostic categories, and (4) to use consistent research methods in all cross-cultural studies.^39^ To be able to design more effective and appropriate prevention/treatment interventions, researchers need to examine variations in the expression and articulation of distress across cultures and to improve definitions and measures of mental illness for diverse populations. The recognition and treatment of common mental disorders through integration of mental health services into primary care is obligatory, and further study of primary care models such as collaborative care will be important. Integrating mental health care into primary care will require high-level training, research on best psychiatric practices, and global ethical norms.

We suggest the optimal approach to building capacity in mental health care around the world will require partnerships between professional resources in HICs and promising health-related institutions, wherever they can be found in LAMICs. The result of these partnerships would be sustainable centers of excellence in mental health that would have the ability to educate in-country primary care physicians and eventually also psychiatrists and child psychiatrists, bachelor's- and master's-level nurses and psychiatric nurses, psychologists at the bachelor's, master's, and doctoral levels, and social workers, all equipped to do discipline-specific mental health supervision and caregiving. Research capabilities will also be essential to establish the epidemiology in country and to do careful outcome measurements of training and interventions. An important goal will be to develop in-country, sustainable clinical care and research capacity.

Once in place, these partners would be able to strengthen educational resources (including faculty syllabi, didactic materials, and infrastructure), promote a new professionalism that uses objective competencies as criteria for classifying health professionals, establish collective planning mechanisms, and deal flexibly with the supply and demand of health professionals for meeting the health needs of local populations. It will also be necessary to move beyond the standard conception of academic centers to an educational model based on academic systems, including the expansive use of primary care settings as vitally important sites for training. The use of networks, alliances, and collaborations between globalized educational institutions, governments, and nongovernmental organizations (including twinning relationships involving HICs and LAMCs) will significantly enhance capacity building, as will nurturing a shared culture of clinical inquiry and of bidirectional intellectual and ethical benefit.

The U.S. National Institute of Health's Medical Education Partnership Initiative recently awarded grants directly to African institutions in a dozen countries, all working in partnership with U.S. medical schools and universities.^41^ The result is a network of about 30 regional partners, country health and education ministries, and more than 20 U.S. and foreign collaborators. The overall goal is to support the training and retention of 140,000 new health workers to improve the capacity of partner countries to deliver primary health care in the field of HIV/AIDS and, to a lesser extent, in maternal and child health and noncommunicable diseases. Unfortunately, although mental health was included in the list of noncommunicable diseases to be addressed, only one linked project among the ten grants for full programs and two for pilot programs involved any connection to capacity building for mental health. While disappointed with the disparity in such support, especially in view of the relative burden of disease, we are nevertheless heartened by such examples of educational *twinning* and look forward to the day when more capacity-building projects for global mental health will be given proper investment.

At MGH, we are seeking support to develop a Mental Health Center of Excellence for Liberia—a country of 3.5 million people, most of whom have been traumatized by decades of civil war. We are confident that we can develop, as a *twinning* partnership with the University of Liberia, sustainable educational systems that will go a long way toward building the necessary professional and lay-caregiver mental health infrastructure that is so sorely needed there. Likewise, we hope that other academic medical centers in HICs will twin with educational centers in LAMICs around the world to develop centers of excellence in mental health. If this movement can catch on, we may be able to appreciably affect worldwide capacity for mental health care and thus, by working together, to reduce the global burden of mental illness.
